# Rectal Alimentation

**Published:** 1878-02-20

**Authors:** 


### RECTAL ALIMENTATION.

Dr. Austin Flint, in *New York Academy of Medicine*, read an interesting paper upon the above subject. The importance of the subject was regarded as sufficient to suggest the question, To whom belongs the credit of having been the first to resort to this method of sustaining nutrition? The author of the paper was not prepared to answer the question. It was Samuel Hood who first suggested it in the present century—in the year 1822. Up to quite a recent date, rectal alimentation had not been regarded as an important measure for sustaining nutrition; at least, only slight reference had been made to it by writers on practical medicine.

Of late, interest in the subject had been somewhat awakened. It had not been altogether because of want of cases, which might show that life could be sustained wholly by rectal alimentation, that such tardiness in recognizing its value had been developed. Reference was then made to a case in which life was sustained for three consecutive months by this means.

A second case was referred to, in which the following clinical facts were made prominent: it was a case of hæmatemesis; exhaustion and exsanguination were very marked. The patient was supported entirely for three weeks by nutritive injections, and the nutritive material was restricted to animal broth, which was tolerated in considerable quantities. Occasional doses of laudanum were added to promote sleep. There was no evacuation from the bowels while rectal alimentation was being pursued. There was a spontaneous evacuation from the bowels, small in quantity, soon after returning to nutrition by the mouth, showing that the nutriment introduced into the bowels had been assimilated.

Reference was made to a third case, in which life was sustained one year and three months by rectal alimentation, and during five years the patient had depended almost entirely upon this method of sustaining nutrition.

Still another case was referred to, in which the patient lived one year under the support chiefly of rectal alimentation.

There were clinical facts sufficient to prove that life could be maintained indefinitely in cases in which recovery was possible, that improvement could be secured in cases in which recovery could not reasonably be expected, and increase in the weight of the body could be realized by rectal alimentation.

The subject was further studied under three heads:

1. Indications for its use.
2. Appropriate diet to be employed for this purpose.
3. Certain practical rules to be observed.

Rectal alimentation was indicated in obstructions of the œsophagus, the cardiac or the pyloric extremity of the stomach, sufficient to prevent adequate nutrition. It was also indicated in the treatment of gastric ulcer, hæmatemesis, acute gastritis, persistent irritability of the stomach, certain cases of typhoid fever, certain cases of coma, etc.

The kinds of aliment best constituted to form rectal diet was regarded as an important question.

The physiology of the subject was briefly considered in this connection, and it was not difficult to understand that, although the aliment met with no digestive juices, the secreting glands, which existed in the large intestine in considerable numbers, might take on a vicarious action when the glands of the stomach and small intestine were not excited into activity by the presence of ingesta.

The idea was also advanced that food introduced into the rectum might excite secretion by the gastric and intestinal glands, and in absence of ingesta in those parts of the alimentary canal, the fluid might pass into the large intestine in sufficient quantity to effect digestion there. Whatever the explanation might be, the clinical fact was well established that digestion of aliment, when placed in the rectum, did take place without the aid of agents which affected digestion outside of the body.

A variety of diet was regarded as better than the persistent use of the same kind of food prepared in precisely the same manner. From analogy, it was reasonable to suppose that such agents as had been found to promote digestion outside of the body might be added to the injections with advantage. Further clinical facts upon that point were needed. The articles now used were meat solution, pancreatic emulsion, Liebig's extract of meat, with or without milk, eggs, mutton and chicken broths. A pancreatic meat emulsion was mentioned, made as follows: From five to ten ounces of finely chopped meat were added to fully one-third of that weight of the fresh pancreas of the ox, the fat being removed, and mixed with about five ounces of water; the whole was reduced to the consistence of a thick soup.

It was desirable to determine more accurately the affections and conditions in which rectal alimentation was most available, and whether the range of that form of diet might not be extended. Experimental observations upon healthy human subjects would be of interest, and were required. In the cases which had fallen under Dr. Flint's observation, the nutritive injection had not been carried above the rectum. In cases in which the rectum was or became irritable, onehalf or a pint of milk could be carried up into the colon and be retained without difficulty. The average quantity of material to be employed in this mode of treatment was from three to six ounces, and the intervals between the injections might vary in length from three to six hours. Small quantities of some preparation of opium might be added to the injection, if they were not well tolerated. It remained to be settled whether or not opium had the same influence upon rectal as upon gastric digestion—namely, to impair it. Preparatory to the beginning of the treatment, the bowels should be emptied either by means of enemata, or by a laxative given by the mouth. As a substitute for drink, when necessary, simple water may be thrown into the bowel, and the surface of the body freely sponged. Alcoholics and medicines might be added to the nutritive injections, or they might be given separately, or they might be used hypodermically. At first, nutritive injections might not be retained, but if persisted in, they would soon be well tolerated. On the other hand, in some cases they were well tolerated at first, but after a time they were not retained. In such cases it was well to stop them for a short time, when they could probably be renewed with success. It was not thought necesssary to wash out the rectum each time prior to using the administration of the nutritive injection. The nutritive injection should be tepid. Firm pressure should be made over the anus with a sponge or towel, until the desire to remove the injection had passed away. If the nutritive injections met the requirements of the case, there would follow a sense of comfort and satisfaction the same as after taking a meal in the ordinary way.—*Medical Record.*

